# A 209 ps Shutter-Time CMOS Image Sensor for Ultra-Fast Diagnosis

**DOI:** 10.3390/s25123835

**Published:** 2025-06-19

**Authors:** Houzhi Cai, Zhaoyang Xie, Youlin Ma, Lijuan Xiang

**Affiliations:** Key Laboratory of Optoelectronic Devices and Systems of Education and Guangdong Province, Shenzhen Key Laboratory of Photonics and Biophotonics, College of Physics and Optoelectronic Engineering, Shenzhen University, Shenzhen 518060, China; hzcai@szu.edu.cn (H.C.); 2300453053@email.szu.edu.cn (Z.X.); 2310455026@email.szu.edu.cn (Y.M.)

**Keywords:** inertial confinement fusion, ultrafast diagnosis, CMOS image sensor, temporal resolution, photodiode

## Abstract

A conventional microchannel plate framing camera is typically utilized for inertial confinement fusion diagnosis. However, as a vacuum electronic device, it has inherent limitations, such as a complex structure and the inability to achieve single-line-of-sight imaging. To address these challenges, a CMOS image sensor that can be seamlessly integrated with an electronic pulse broadening system can provide a viable alternative to the microchannel plate detector. This paper introduces the design of an 8 × 8 pixel-array ultrashort shutter-time single-framing CMOS image sensor, which leverages silicon epitaxial processing and a 0.18 μm standard CMOS process. The focus of this study is on the photodiode and the readout pixel-array circuit. The photodiode, designed using the silicon epitaxial process, achieves a quantum efficiency exceeding 30% in the visible light band at a bias voltage of 1.8 V, with a temporal resolution greater than 200 ps for visible light. The readout pixel-array circuit, which is based on the 0.18 μm standard CMOS process, incorporates 5T structure pixel units, voltage-controlled delayers, clock trees, and row-column decoding and scanning circuits. Simulations of the pixel circuit demonstrate an optimal temporal resolution of 60 ps. Under the shutter condition with the best temporal resolution, the maximum output swing of the pixel circuit is 448 mV, and the output noise is 77.47 μV, resulting in a dynamic range of 75.2 dB for the pixel circuit; the small-signal responsivity is 1.93 × 10^−7^ V/e^−^, and the full-well capacity is 2.3 Me^−^. The maximum power consumption of the 8 × 8 pixel-array and its control circuits is 0.35 mW. Considering both the photodiode and the pixel circuit, the proposed CMOS image sensor achieves a temporal resolution better than 209 ps.

## 1. Introduction

Inertial confinement fusion (ICF) represents a cutting-edge research area in contemporary physical science, where the fusion reaction of deuterium–tritium plasma is achieved under extreme conditions through the interaction between high-energy lasers or secondary X-ray radiation and microtarget pellets. The ICF process generates significant radiation across multiple spectral bands. By imaging high-temperature and high-density plasma in the implosion compression region using an optical system, dynamic images of implosion compression and other critical information can be obtained. The key physical phenomena occurring during this process take place on a spatial scale of micrometers and a temporal scale of nanoseconds, presenting a dual challenge to diagnostic techniques: achieving sub-micrometer spatial resolution and picosecond-level temporal resolution accuracy [1,2].

Typical ultrafast diagnostic devices commonly used in ICF and related fields include framing cameras, streak cameras, and compressive sensing ultrafast imaging systems [3]. Framing cameras are capable of high spatiotemporal resolution two-dimensional imaging and can be categorized into microchannel plate (MCP), electron pulse broadening [4,5], all-solid-state [6], and all-optical solid-state [7] framing techniques. Within the ICF diagnostic technology system, framing cameras, as core diagnostic equipment, have undergone significant technological advancements. Early MCP-based framing systems achieved a temporal resolution of 60–100 ps, whereas the introduction of electron pulse broadening improved this resolution to 4 ps [8]. Although these cameras satisfy the requirements for temporal resolution, further development of ICF research has highlighted the need to address their large size and reliance on high-voltage signals for operation. To acquire continuous images, these systems are often coupled with pinhole imaging arrays or Kirkpatrick–Baez (KB) microscopes, which generate multiple images of different regions of the MCP microstrip cathode [9,10]. However, owing to the geometric distortion caused by multifield-of-view imaging, achieving precise single-line-of-sight (SLOS) reconstruction remains challenging [11]. With advancements in semiconductor technology, all-solid-state framing technology based on CMOS processes has demonstrated significant advantages. Its integrated design not only overcomes the volume limitations of traditional systems but also achieves critical breakthroughs in SLOS imaging [12,13].

In 2008, Robert Berger et al. developed a small-sized CMOS image sensor test chip for rapid photography [14], with a pixel-array size of 64 × 64 and an adjustable exposure time ranging from 75 to 305 ps. Based on this chip, the research team from Lawrence Livermore National Laboratory (LLNL) in the United States proposed a 512 × 512 pixel-array Read Out Integrated Circuit (ROIC) in 2012, which adopted an extended H-tree structure and achieved quad-split imaging [15]. In 2015, Mochizuki Futa et al. from Shizuoka University in Japan developed a 32-split CMOS image sensor capable of continuous shooting at 200 million frames per second [16]. In 2016, the Ultrafast Diagnostics Team at Shenzhen University developed a 40 × 48 pixel-array CMOS image sensor based on a 0.5 μm CMOS process, with a minimum shutter time of 75 ps, which is applicable for single-frame imaging [17]. The “Icarus” system developed by Sandia National Laboratories (SNL) in the United States in 2017 increased the temporal resolution to the 1–2 ns level [18,19]. By coupling with the electron beam time-stretching technology, the LLNL laboratory successfully developed a quad-split X-ray imaging system with a temporal resolution of 30 ps and a spatial resolution of 35 μm [20]. In 2021, Hui Chen et al. from the LLNL laboratory integrated two “Icarus” systems onto the imaging device G-LEH-2, enhancing the sensitivity to low-energy photons and the working ability under high-radiation conditions, and achieving octuple-split imaging [21]. The “Daedalus” system launched in 2023 achieved a full-well capacity of 1.5 Me^−^, a pixel sensitivity of 9.58 × 10^−7^ V/e^−^, and an image uniformity greater than 95% [22].

To further enhance the temporal resolution capability of CMOS image sensors in a single exposure, this article presents a comprehensive design of an ultrashort shutter-time CMOS circuit. The proposed 8 × 8 pixel-array CMOS image sensor employs a stacked structure [23], which is based on the silicon epitaxial process and the 0.18 μm standard CMOS process. A PIN-type photodiode is designed using the silicon epitaxial process, and its spectral response and temporal resolution performance are evaluated. Additionally, a 5T pixel circuit with a supply voltage of 1.8 V and its associated control circuits are designed based on the 0.18 μm standard CMOS process, and the ultimate temporal resolution capability along with other performance metrics are simulated under a single-frame structure.

## 2. CMOS Image Sensor Design Methodology

**Figure 1:**
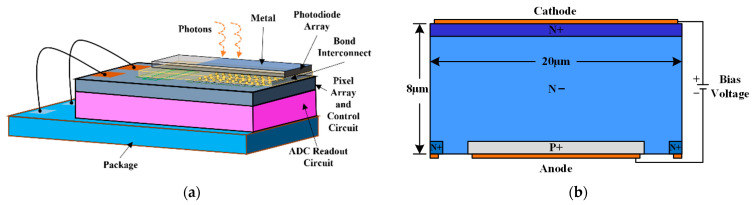
(**a**) Schematic diagram of the overall structure of the CMOS image sensor; (**b**) Schematic illustration of the structure of the PIN-type silicon-based photodiode.

The ultra-short shutter-time single-frame CMOS image sensor proposed in this work employs a 3D-IC stacked structure, as illustrated in Figure 1a for its structural principle. The top layer of the CMOS image sensor comprises a photodiode array covered by a metal layer. Beneath the photodiode array lies the pixel array and its associated control circuitry. These two components are interconnected by hybrid bonding technology. Moreover, the ADC readout circuit module resides beneath the pixel array, and the two parts are connected by through-silicon vias (TSV). With this 3D-IC stacked architecture, the advantages of different fabrication processes can be fully leveraged, and crosstalk among various circuit modules can be effectively minimized. This paper focuses primarily on detailing the design and performance of the photodiode and pixel-array circuit portion of the sensor.

In CMOS image sensors, the photodiode (PD) functions as the key photosensitive component. Serving as the photoelectric converter in CMOS image sensors, its performance directly influences the sensitivity, speed, and reliability of the imaging system.

The specific structure of the photodiode was designed using the semiconductor process in conjunction with the device simulation software Silvaco TCAD 2021. The structure of the photodiode is shown in Figure 1b. A vertically distributed PIN silicon-based photodiode was employed as the fundamental detection unit. When the photodiode is irradiated by a light signal, it generates electron-hole pairs. These carriers are collected under the influence of the reverse bias voltage applied to the semiconductor material and subsequently transferred to the storage capacitor within the pixel unit circuit. The PIN silicon-based photodiode is structurally similar to a conventional PN junction diode but includes an additional near-intrinsic region between the two heavily doped regions. Under reverse bias operation, the entire intrinsic region becomes fully depleted, resulting in higher detection efficiency in the visible light band compared to the standard PN junction photodiode [24].

**Figure 2:**
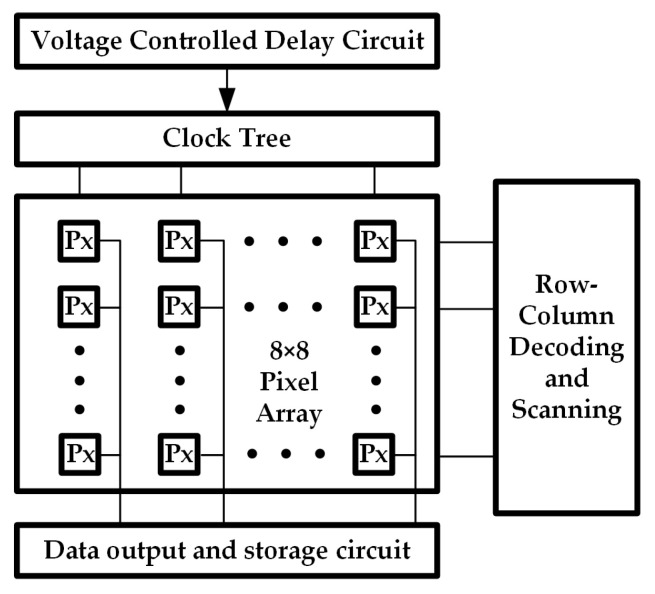
Schematic illustration of the circuit structure of a CMOS image sensor.

In each pixel unit of the CMOS image sensor pixel array, the photodiode collaborates with other transistors in the pixel circuit, including the reset transistor, and source follower. During the exposure period, the photodiode continuously accumulates photogenerated charges, the quantity of which is proportional to the incident light intensity and the exposure time. The accumulated charges are then read out in the form of voltage one-by-one through the source follower and the gating transistor. The circuit structure of the 8 × 8 pixel-array CMOS image sensor designed in this paper is illustrated in Figure 2, encompassing the pixel array, voltage-controlled delay circuit (VCD), clock tree, and row-column decoding and scanning circuits.

**Figure 3:**
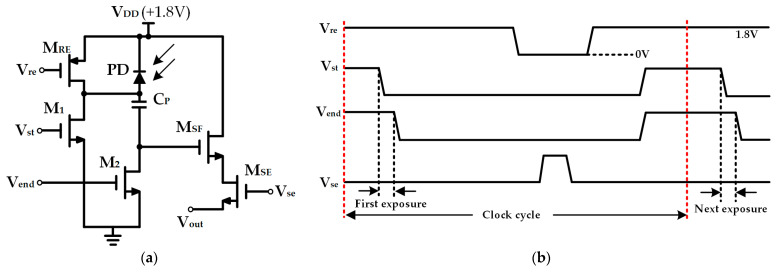
Structural diagram of the pixel and signal timing diagram: (**a**) Pixel circuit; (**b**) Signal timing diagram.

The pixel unit circuit of the CMOS image sensor designed in this paper employs a 5T pixel structure, as illustrated in Figure 3a. Herein, PD denotes the PIN-type photodiode designed in this research; C_P_ is the storage capacitor for the pixel exposure signal. M_1_ and M_2_ control the start and end of exposure, respectively, and both are designed as ring-gate NMOS transistors, with the drain located inside the ring gate. Compared to the fork-gate NMOS transistors, the ring-gate NMOS transistors have a smaller drain capacitance. Moreover, due to the smaller drain area and being surrounded by the source, the proportion of photogenerated electrons collected by the drain of the ring-gate NMOS transistors in the substrate of the integrated circuit is smaller than that collected by the drain of the fork-gate NMOS transistors of the same size. Therefore, a higher shutter efficiency can be achieved. M_RE_ serves as the reset transistor, and transistors M_SF_ and M_SE_ are used for signal buffering and gating. V_re_ is the reset signal, V_st_ and V_end_ are the control signals for the start and end of exposure, respectively, V_se_ is the output gating signal, and V_out_ is the output voltage signal.

The timing diagram of the pixel unit circuit illustrated in Figure 3a is shown in Figure 3b, and its working process is as follows: Prior to the exposure, V_re_ is set to ground to perform the reset operation of the circuit. Then, V_re_, V_st_, and V_end_ are set to V_DD_, and V_se_ is set to ground. At this moment, M_1_ and M_2_ are conducting, whereas M_RE_ and M_SE_ are nonconducting. The current signal corresponding to the incident light on the PD flows to ground through M_1_. When the exposure commences, V_st_ transitions to ground, M_1_ becomes nonconducting, and the current generated by the PD charges the C_P_. After a certain period, V_end_ transitions to ground, and the exposure concludes. All transistors are in a nonconducting state. The charge stored in the C_P_ remains unchanged, and the pixel exposure process ends. The time difference from the start to the end of the exposure is the exposure time for signal acquisition and imaging. After the exposure ends, V_re_ is pulled down to ground, and M_RE_ is conducting, pulling the anode of the photodiode PD to V_DD_. At this point, a voltage signal ready for readout forms on the gate of M_SF_. Subsequently, V_se_ can be set to V_DD_, at which time, M_SE_ is conducting, and the signal within the pixel can be output. After the signal is read out, the M_RE_, V_st_, V_end_, and V_se_ signals are controlled to make M_SF_ and M_SE_ nonconducting and M_RE_ conducting. The pixel unit circuit is reset once again and awaits the next signal acquisition and readout.

**Figure 4:**
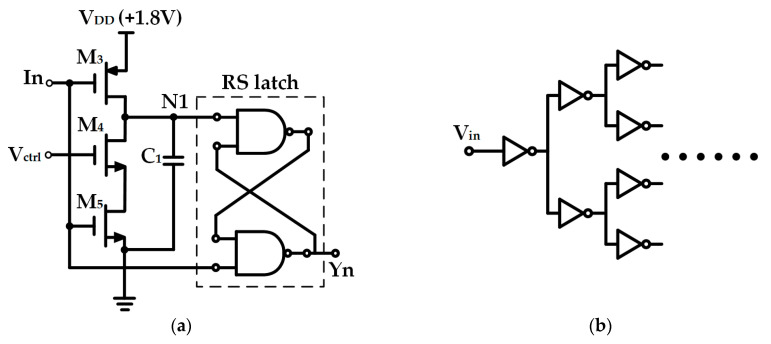
Structural diagram of control circuits: (**a**) Voltage-controlled delay circuit; (**b**) Clock tree circuit.

The VCD circuit, as illustrated in Figure 4a, is employed to generate the signals that control the start and end of exposure and can regulate the exposure time through controlling the voltage. It consists of a PMOS transistor M_3_, NMOS transistors (M_4_ and M_5_), a capacitor C_1_, and an RS flip-flop. Herein, In represents the trigger-on signal for the control delayer, V_ctrl_ denotes the gate voltage control signal of M_4_, and Yn is the output signal. The working principle of the signal delay of VCD is as follows: By varying the resistance in the circuit, the discharge rate of capacitor C_1_ can be modified. The RS latch is capable of preserving the voltage variation at node N1, thereby outputting the delayed signal. Prior to triggering, the signal In is at ground. At this moment, the input nodes N1 of the RS flip-flop is at V_DD_, and the RS flip-flop is reset, with the output signal Yn at V_DD_. After triggering, the voltage of signal In rises. At this time, both the input node N1 and In of the RS flip-flop are at V_DD_, the output of the RS flip-flop remains temporarily unchanged, and the voltage at node N1 gradually decreases. Its descent rate can be controlled within a certain range by V_ctrl_. After a certain period, when the voltage at node N1 drops to a level sufficient to trigger the RS flip-flop, the RS flip-flop is set, and the output signal Yn rapidly decreases, thereby achieving the effect of signal delay.

The clock tree circuit adopts a binary clock tree structure. It is capable of synchronously allocating the V_st_ and V_end_ signals generated by the VCD circuit into 64 channels and distributing them to 64 pixel-units of the 8 × 8 pixel-array, as illustrated in Figure 4b. It is employed for precise clock signal distribution, synchronizing the start and end times of exposure across all the pixels in the pixel array. This prevents uneven brightness between rows or columns due to timing deviations and minimizes signal crosstalk.

**Figure 5:**
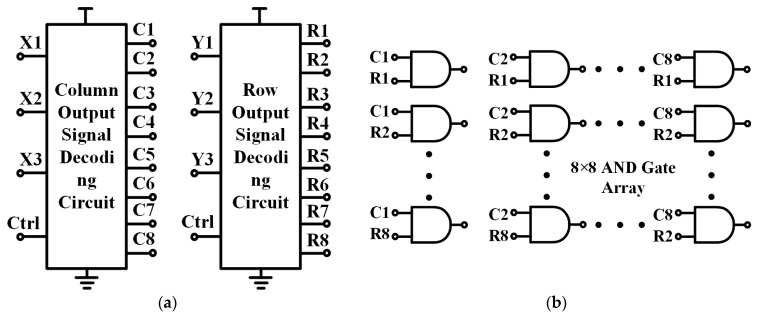
Row-column decoding and scanning circuit: (**a**) Row-column decoding circuit; (**b**) AND gate array.

The row-column decoding and scanning circuit is employed to furnish the V_se_ gating signal, and its configuration is presented in Figure 5. The output of each gate in the AND gate array corresponds to the gating signal V_se_ of one pixel. The row decoder and the column decoder obtain eight distinct output voltages by inputting different combinations of true-value voltages. By combining the output voltages of the two decoders and inputting them into the AND gate array, 64 sets of output signals can be acquired, which are subsequently utilized to output the output voltages of each pixel element in the pixel array.

## 3. Results

### 3.1. Performance Test of the Photodiodes

**Figure 6:**
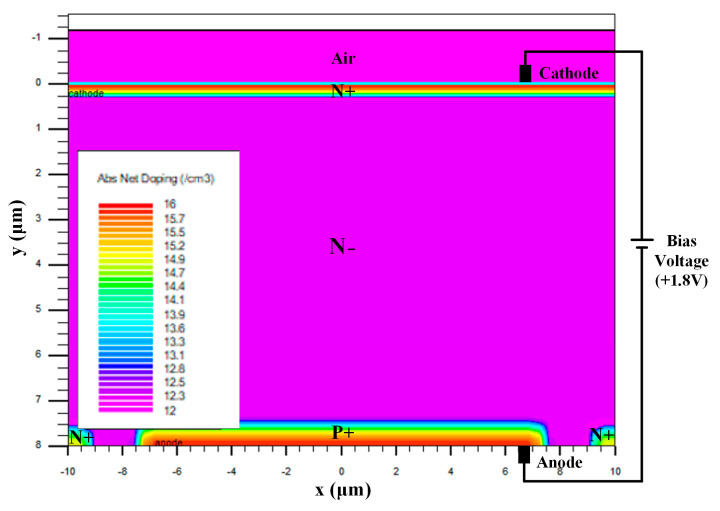
Simulation structure diagram of PIN photodiode.

The designed PIN-type photodiode underwent process simulation using the Atlas module of the semiconductor process and device simulation software Silvaco TCAD 2021, as shown in Figure 6. During the simulation, the PIN-type photodiode was reverse-biased at 1.8 V.

**Figure 7:**
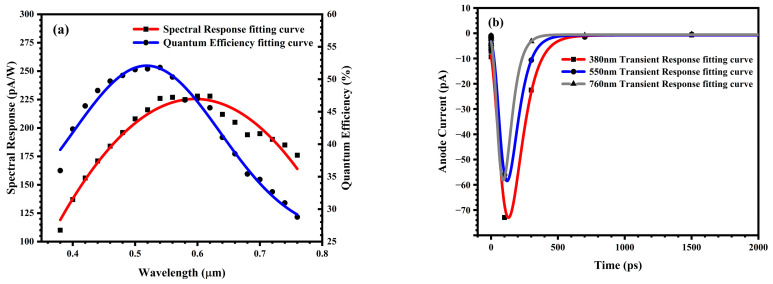
(**a**) Spectral response characteristic curve and quantum efficiency of PIN-type photodiodes to visible light; (**b**) Transient response characteristics.

To simulate the spectral response characteristics of the photodiode to visible light in this study, during the simulation, visible light of different wavelengths ranging from 380 nm to 760 nm at an intensity of 1 W/cm^2^ was used to irradiate the photodiode above the N+ region of the photodiode. By recording the photocurrent flowing through the anode, the spectral response characteristic curve of the photodiode was obtained, as illustrated in Figure 7a.

The conversion relationship between the quantum efficiency (QE) and the spectral responsivity (SR) of a photodiode can be expressed as:

(1) $$\mathrm{SR}\left(\lambda \right)=\frac{I(\lambda )}{P(\lambda )}=\frac{q}{h\nu }\mathrm{QE}\left(\lambda \right)=\frac{\lambda }{1240}\mathrm{QE}(\lambda )$$

where I represents the magnitude of the photocurrent, P denotes the incident light power, q is the elementary charge, h is Planck’s constant, ν is the frequency of light, and λ is the wavelength.

Based on Equation (1) and the spectral response characteristic curve of visible light, the QE of the photodiode can be calculated, as illustrated in Figure 7a. The QE of this photodiode within the visible light range is above 30%.

To evaluate the temporal resolution capability of the designed PIN-type photodiode, pulsed light with different wavelengths was applied for irradiation at a position 1 μm directly above the photodiode. The pulsed light had an intensity of 1 W/cm^2^ and a pulse width of 1 ps. The current flowing through the anode of the photodiode was recorded, and the simulation results of the transient response characteristics of the PIN-type photodiode under irradiation with light of different wavelengths are presented in Figure 7b. Herein, the time-resolution capability of the photodiode is defined as the full width at half maximum (FWHM) of the transient response curve of the photocurrent induced by sufficiently short and weak pulsed light in a reverse-biased photodiode. The time-resolution capability for light with a wavelength of 380 nm is 196 ps, that for 550 nm light is 180 ps, and that for 760 nm light is 162 ps. It can be concluded that for any visible-light wavelength, the time-resolution capability T_1_ of the PIN-type photodiode fabricated using the silicon epitaxial process in this study is consistently better than 200 ps.

### 3.2. Performance Test of the Pixel Circuit

#### 3.2.1. Analysis of the Theoretical Optimal Time Resolution Capacity of Pixel Circuit

**Figure 8:**
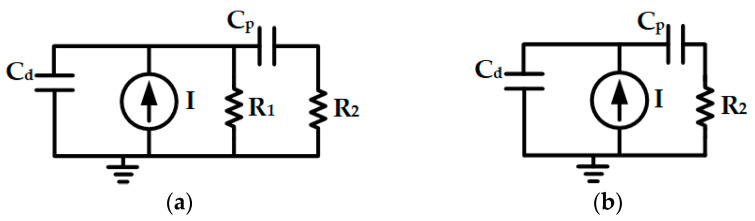
Simplified circuit model of a pixel circuit: (**a**) Before exposure; (**b**) During exposure.

To evaluate the theoretical temporal resolution capability determined by the pixel circuit, the pixel circuit model is simplified both before the onset of exposure and during the exposure process, as illustrated in Figure 8.

Figure 8a shows the simplified circuit model of the pixel unit circuit while awaiting the trigger prior to exposure. At this point, both transistors M_1_ and M_2_ in the pixel unit circuit shown in Figure 3a are in the conducting state. Capacitor C_d_ primarily consists of the junction capacitance of the photodiode PD and encompasses the capacitances from transistors M_1_ and M_RE_. Capacitor C_p_ is the sampling capacitor. Resistors R_1_ and R_2_ represent the on-resistances of transistors M_1_ and M_2_, respectively. The pulse current source I is assumed to generate a pulse current with a total charge of Q_P_ that is short enough before exposure, and the time difference between the moment when this pulse current is emitted and the start of exposure is t_1_. Let Q_1_ be the charge stored on capacitor C_p_ after the exposure commences and the shutter remains open for a sufficiently long duration. To simplify the calculation process, assume C = C_d_ = C_p_. By applying the Laplace transform method to conduct circuit analysis on the aforementioned simplified model, the expression for Q_1_ can be derived as:

(2) $$Q_{1}=\frac{\left(2R_{1}-R_{2}\right)\sin h\left(\tau t_{1}\right)+2R_{1}R_{2}C\tau \cdot \cos h(\tau t_{1})}{4R_{1}R_{2}C\tau }\cdot e^{-\frac{2R_{1}+R_{2}}{2R_{1}R_{2}C}t_{1}}\cdot Q_{p}$$

(3) $$\tau =\frac{\sqrt{4R_{1}^{2}+R_{2}^{2}}}{2R_{1}R_{2}C}$$

In the pixel unit circuit of the CMOS image sensor proposed in this work, when the supply voltage is 1.8 V, R_1_ = R_2_ = 2.09 kΩ, and the capacitance C is approximately 15.54 fF. As t_1_ increases, Q_1_ decreases. Let t_half1_ denote the value of t_1_ when Q_1_ decays to half of its maximum value. t_half1_ is 34.3 ps.

Figure 8b shows the simplified circuit model of the pixel unit circuit during exposure after triggering. At this stage, transistor M_1_ has been turned off, whereas transistor M_2_ remains in the conducting state. Assume that the current source I generates a pulse current with a total charge of the Q_P_ that is short enough when the shutter is open. Let t_2_ represent the elapsed time after the emission of the pulse current. The accumulated charge on capacitor C_p_ at time t_2_ is denoted as Q_2_. By applying the Laplace transform method for circuit analysis, the expression for Q_2_ can be derived as:

(4) $$Q_{2}=\frac{C_{1}}{C_{1}+C_{d}}[1-e^{-\frac{C_{1}+C_{d}}{R_{2}C_{1}C_{d}}t_{2}}]Q_{p}$$

Let t_half2_ denote the time elapsed when Q_2_ increases to half of its maximum value after the emission of the aforementioned pulse current, then t_half2_ can be expressed as:

(5) $$t_{\mathrm{half}2}=\ln(2)\frac{R_{2}C_{1}C_{d}}{C_{1}+C_{d}}$$

For the CMOS image sensor designed in this study, C_p_ = 15.54 fF and C_d_ = 159.6 fF. When the supply voltage is 1.8 V and R_2_ = 2.09 kΩ, t_half2_ is calculated to be 20.5 ps. Consequently, the theoretical optimum temporal resolution capability of the pixel unit circuit designed herein is t_half1_ + t_half2_ = 54.8 ps.

#### 3.2.2. Simulation of the Pixel Circuit

To evaluate the actual optimal temporal resolution capability of the circuit structure designed in the analog simulation, an 8 × 8 pixel-array circuit is simulated using a pulsed current source. An 8 × 8 pulsed current source signal array is provided for the pixel array, where the magnitude of each pulsed current source signal is set to 500 µA, the pulse width is 10 ps, both the rising edge and falling edge durations are 5 ps, and the interval between the peaks of adjacent pulsed current sources is 5 ps. Consequently, a time-measuring scale with a range of 0–315 ps and a minimum resolution of 5 ps can be established. The row-column decoding scanning circuit shown in Figure 5 is utilized to provide the required V_se_ gating signal for each pixel.

**Figure 9:**
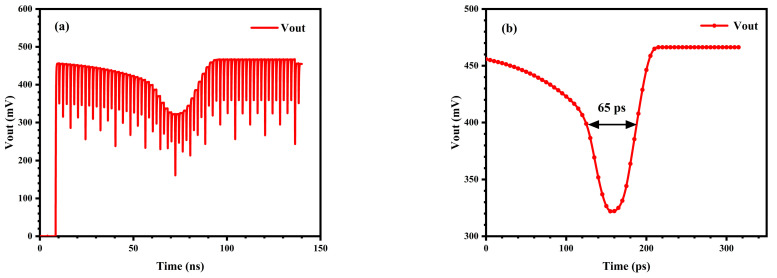
(**a**) Output voltage signal curve of the sensor of the 8 × 8 pixel-array at an exposure time of 54.8 ps; (**b**) Temporal resolution curve.

The V_ctrl_ of the VCD circuit was set to 1.17 V, and the exposure time of the pixel array of the CMOS image sensor was set to the theoretical optimal temporal resolution of 54.8 ps. The output voltage of the 8 × 8 pixel-array, measured by sequentially scanning and reading out each pixel unit through 64-channel V_se_ signals, is illustrated in Figure 9a. The descending portion of the scan signal curve in the figure indicates that the pulse current source signal was detected within the exposure time, which corresponds to the exposure instant of this sensor.

The scanning gate output signal curve in Figure 9a was transformed into a temporal resolution curve within the exposure time, characterized by the pulsed light source array, as shown in Figure 9b. The FWHM of the curve in the figure represents the temporal resolution capability of the image sensor under this exposure condition, which is 65 ps, which is slightly inferior to the theoretically calculated value. This includes the influence of the falling edge of the exposure control signal.

**Figure 10:**
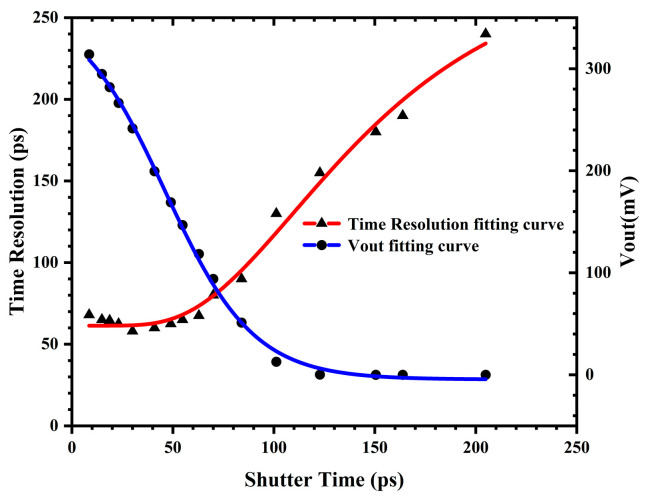
The temporal resolution capabilities and output voltages of sensor pixel circuits at different shutter times and for a 500 µA pulse current source input.

While maintaining the input pulse current source at 500 µA, by adjusting the exposure time, the temporal resolution capability and output voltage of the sensor pixel circuit under different shutter times were simulated, as illustrated in Figure 10. The temporal resolution capability decreases with decreasing shutter time. When the shutter time becomes excessively small, the temporal resolution capability of the sensor tends to stabilize. The optimal temporal resolution capability T_2_ is approximately 60 ps. Considering the combined influences of both the photodiode and the pixel circuit on the temporal resolution capability of the sensor, the temporal resolution capability T of the CMOS image sensor designed in this study can be expressed by the following equation:

(6) $$T=\sqrt{T_{1}^{2}+T_{2}^{2}}$$

T_1_ is better than 200 ps, while T_2_ is 60 ps. Therefore, the result is that T is greater than 209 ps.

**Figure 11:**
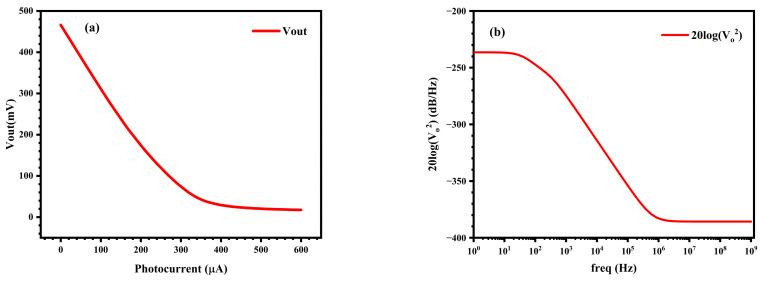
(**a**) Relationship between the output voltage and the input photocurrent when the shutter time is 40 ps; (**b**) Power spectral density of the output noise of the pixel circuit.

Set the V_ctrl_ of the VCD circuit to 1.25 V; under the exposure condition with an optimal temporal resolution capability of 60 ps, the relationship between the output voltage of the pixel circuit and the input photocurrent is illustrated in Figure 11a. As the input photocurrent increases, the output voltage tends to saturate. When there is no photocurrent input, the output V_dark_ of the pixel circuit is 466 mV, with a maximum output voltage swing of approximately 448 mV and a linear output range from 0 to 180 μA. The noise simulation analysis of the circuit at the optimal temporal resolution capability yields the power spectral density curve of the output noise, as shown in Figure 11b, and the total equivalent output noise is determined to be 77.47 μV. At low frequencies, it is mainly 1/f noise, while at high frequencies, it is mainly thermal noise. The expression for the dynamic range (DR) is:

(7) $$\mathrm{DR}=20\log\left(\frac{{\left|V_{\mathrm{out}}-V_{\mathrm{dark}}\right|}_{\max}}{V_{\mathrm{noise}}}\right)\;[\mathrm{dB}]$$

Among them, |V_out_ − V_dark_|_max_ represents the maximum output swing of 448 mV of the sensor at its optimal temporal resolution capability, and V_noise_ denotes the output noise of 77.47 μV. Consequently, the dynamic range can be calculated to be 75.2 dB.

In order to acquire the small-signal responsivity of the image sensor described in this paper, the difference in the total current flowing through the power supply of the pixel circuit was measured under two conditions: when there was zero input photocurrent and when the input photocurrent was 100 μA. During this simulation process, the pixel remained in a state of waiting for exposure. Subsequently, by the difference in the output voltage of the pixel circuit under these two input photocurrent conditions, the small-signal responsivity of the designed sensor was determined to be 1.93 × 10^−7^ V/e^−^. Moreover, based on the output swing of 448 mV, the full-well capacity of the designed pixel circuit was calculated to be 2.3 Me^−^.

In the designed 5T pixel circuit, the power consumption of M_SF_ and M_SE_ transistors constitutes the majority of the total power consumption, primarily due to energy dissipation during the charging and discharging processes of the sampling capacitor C_p_. When M_SE_ is turned on, the charge accumulated on the sampling capacitor C_p_ during the exposure period is discharged, with the energy consumption during charging and discharging being:

(8) $$W=C_{P}V_{\mathrm{DD}}V_{\mathrm{read}}$$

The voltage value V_read_ across the sampling capacitor C_p_ after completion of the sampling process is the output value of the pixel unit following photogenerated charge transfer. This value decreases with increasing light intensity. According to Equation (8), the energy consumption of the 5T pixel unit within one operational cycle consequently decreases as environmental illumination intensifies.

**Figure 12:**
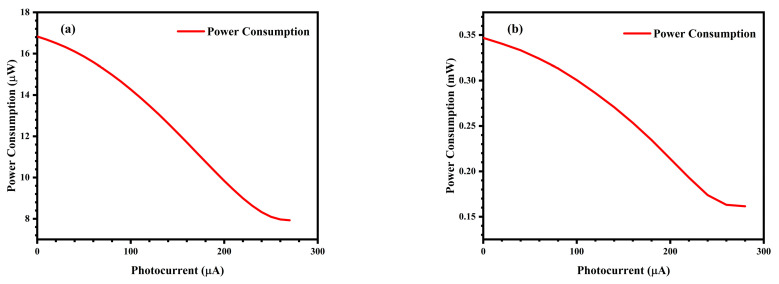
(**a**) Relationship between the power consumption of the single-pixel unit and the input photocurrent; (**b**) Relationship between the power consumption of 8 × 8 pixel-array and its control circuits and the input photocurrent.

As shown in Figure 12a, the simulated power consumption results demonstrate that the single-pixel circuit’s power consumption decreases with increasing input photocurrent, with the maximum power consumption being 16.8 μW. The power consumption simulation results of the 8 × 8 pixel-array and its control circuits is shown in Figure 12b, and the maximum power consumption is 0.35 mW. Due to the global sharing of the control circuit (such as the VCD) in the pixel array, the overall power consumption of an 8 × 8 pixel-array is lower than 64 times the power consumption of a single-pixel unit.

The characteristics and simulation results of the designed image sensor and a comparison with prior works are summarized in Table 1.

## 4. Conclusions

This article presents an 8 × 8 pixel-array single-frame CMOS image sensor with an ultra-short shutter time. The PIN-type photodiode is fabricated via a silicon epitaxial process. The pixel and its control circuits are implemented based on 0.18 μm standard CMOS technology, including a 5T structure pixel unit, a voltage-controlled delay device, a clock tree, and row-column decoding and scanning circuits. The simulation results show that under a bias voltage of 1.8 V, the photodiode achieves a quantum efficiency exceeding 30% in the visible light band, with a temporal resolution for visible light greater than 200 ps. The shortest temporal resolution capability of the pixel circuit is 60 ps, the maximum output swing is 448 mV, the output noise is 77.47 μV, the dynamic range reaches 75.2 dB, the small-signal responsivity is 1.93 × 10^−7^ V/e^−^, the full-well capacity is 2.3 Me^−^, and the maximum power consumption of the 8 × 8 pixel-array and its control circuits is 0.35 mW. By comprehensively considering the contributions of both the photodiode and the pixel circuit, the temporal resolution capability of the designed ultra-short shutter-time single-frame CMOS sensor is better than 209 ps. Subsequent research will focus on designing the ADC readout circuit and designing the layout for this CMOS image sensor, followed by tape-out testing. The architectural feasibility of this CMOS sensor configuration in large pixel arrays will be further explored. For instance, larger clock tree circuits may introduce temporal synchronization errors when distributing shutter control signals to each pixel units, while complex circuit layouts could exacerbate signal crosstalk effects. These factors may potentially affect the sensor’s temporal resolution and readout noise. Therefore, subsequent research will pay more attention to the rationality of the layout design and optimize the circuit design to reduce the above-mentioned influences to finally obtain a large array CMOS image sensor with a time resolution of less than 100 ps and a spatial resolution of 20 μm.

## Figures and Tables

**Table 1:** Comparison with previous ultra-fast gated CMOS image sensors.

Reference	Robert Berger [14]	Fan Zhang [17]	Looker Quinn [22]	This Work
Supply voltage	1.8 V	5 V	5 V	1.8 V
Process	0.18-μm CMOS	0.5-μm CMOS	0.35-μm CMOS	0.18-μm CMOS
Resolution	64 × 64 pixels	40 × 48 pixels	1024 × 512 pixels	8 × 8 pixels
Power consumption	125 mW	50 mW	-	0.35 mW
Full capacity	0.7 Me^−^	1.2 Me^−^	1.5 Me^−^	2.3 Me^−^
Small-signal responsivity	1.1 × 10^−7^ V/e^−^	1.47 × 10^−6^ V/e^−^	9.58 × 10^−7^ V/e^−^	1.93 × 10^−7^ V/e^−^
Output swing	0.8 V	1.8 V	1.4 V	448 mV
Shortest shutter time	200 ps	75 ps	1 ns	209 ps

## Data Availability

Data are contained within this article.
